# Free fillet upper extremity flap reconstruction after forequarter amputation: A systematic review and meta-analysis

**DOI:** 10.1016/j.jpra.2026.08.009

**Published:** 2026-08-20

**Authors:** Joshua Khorsandi, Liahm Blank, Jake Patrick Young, Parker Lehmann, Abu-Bakr Ahmed, Brian Mansoury, Aria Damavandi, Demitri Franzoni, Joshua MacDavid

**Affiliations:** a Kirk Kerkorian School of Medicine at University of Nevada Las Vegas, Las Vegas, NV 89106, USA; b Department of Plastic and Reconstructive Surgery, University of Nevada Las Vegas, Las Vegas, NV 89106, USA

**Keywords:** Forequarter amputation, Spare parts surgery, Free flap, Chest wall reconstruction, Microsurgery

## Abstract

**Background:**

Forequarter amputation and related shoulder-girdle ablative procedures create extensive defects, often in previously irradiated fields. “Spare-parts” reconstruction using tissue from the amputated limb, most commonly the free fillet upper-extremity flap (FFUE), offers donor-site–sparing coverage. The reliability and complication profile of FFUE reconstruction remain uncertain because evidence is limited to small series and case reports.

**Methods:**

A PRISMA 2020–compliant systematic review and meta-analysis was conducted. PubMed, Scopus, and Embase were searched on 25 Nov 2025 using prespecified strategies, yielding 404 records; after deduplication and screening, 17 studies were included. We included studies reporting microvascular transfer of an upper-extremity fillet flap (forearm/arm/hand, with or without bone) to reconstruct defects created by forequarter amputation, interscapulothoracic amputation, or shoulder disarticulation. Primary outcomes were complete flap survival, any postoperative complication, and reoperation. Random-effects meta-analyses of proportions were performed using a generalized linear mixed model (GLMM) with logit link; τ and prediction intervals were reported. Sensitivity analyses used inverse-variance pooling with Hartung–Knapp adjustment.

**Results:**

Seventeen studies encompassing 44 patients met inclusion criteria. Most reconstructions followed forequarter amputation (86.4%) for malignant disease, frequently after prior radiotherapy and/or chemotherapy (75.0%). The pooled rate of complete flap survival was 97.0% (95% CI 88.8–99.3), with no reported total flap losses and one case of partial distal necrosis (2.3%). Any postoperative complication occurred in 31.2% (95% CI 18.7–47.3), and reoperation in 26.0% (95% CI 14.6–42.0). The most common complications were necrosis (16.1%), thrombosis (6.8%), and wound dehiscence (6.9%). Despite substantial heterogeneity and frequent prior multimodal therapy, free fillet upper-extremity flaps demonstrated consistently high reliability across reported series.

**Conclusions:**

FFUE reconstruction shows near-universal survival despite extreme defects and frequent prior multimodal oncologic therapy. Complications and reoperations are not rare but appear acceptable in context. Standardized reporting and multi-institutional collaboration are needed to define best practices and to evaluate patient-centered outcomes.

## Introduction

Forequarter amputation (FQA) and related shoulder-girdle ablative procedures, such as interscapulothoracic amputation and shoulder disarticulation, remain necessary in select patients with locally advanced malignancy, uncontrolled infection, or catastrophic trauma. These operations are among the most morbid in oncologic and trauma surgery, producing extensive composite defects involving the shoulder, axilla, chest wall, and proximal arm.^1^ Coverage is often required over exposed pleura or lung, vascular repairs, prosthetic mesh, clavicular/rib resections, or large dead spaces.^2^ The reconstructive goal is not cosmetic alone; durable closure protects intrathoracic structures, reduces infection and wound breakdown, and can facilitate timely adjuvant therapy.

Conventional reconstruction includes regional pedicled flaps (latissimus dorsi, pectoralis major, rectus abdominis variants) and free tissue transfer from a distant donor site (e.g., anterolateral thigh).^3^ In many FQA patients, however, these options are constrained by prior surgery, radiotherapy, compromised recipient vessels, and limited physiologic reserve.^4^ Chest wall reconstruction principles emphasize replacing “like with like,” providing robust vascularized tissue to cover exposed critical structures and obliterate dead space, particularly in irradiated fields, because marginal perfusion and dead space strongly predispose to infection and dehiscence.

The “spare parts” paradigm offers a distinctive solution: if distal tissues of the amputated limb are oncologically clear (or viable in trauma), they can be recycled into a fillet flap. The free fillet upper-extremity flap (FFUE) converts the amputated arm/forearm into a microsurgical flap with large-caliber inflow/outflow, providing immediate coverage without a second donor site. Technical descriptions highlight additional advantages: access to large recipient vessels (e.g., subclavian system) and the possibility of incorporating substantial muscle to fill dead space. In selected patients, FFUE transfer has also been combined with targeted muscle reinnervation (TMR) to mitigate neuroma/phantom pain and create muscle targets for future prosthetic control.

Despite these theoretical benefits, FFUE reconstruction remains uncommon and is reported mainly as case reports and small series. Consequently, surgeons lack consolidated estimates of flap reliability, complication rates, and reoperation burden. The objective of this review was to synthesize current evidence and quantify outcomes through meta-analysis.

## Methods

### Protocol and reporting

A review protocol was developed a priori to define the research question, eligibility criteria, outcomes of interest, data extraction strategy, and planned statistical analyses. The conduct and reporting of this systematic review and meta-analysis adhered to the Preferred Reporting Items for Systematic Reviews and Meta-Analyses (PRISMA) 2020 guidelines.

Reporting completeness of included studies was cross-checked against relevant STROBE domains when applicable, in accordance with journal guidance for observational surgical research. Protocol registration was performed. (https://osf.io/k3e2y)

### Eligibility criteria

We included clinical studies of any design (case reports, case series, cohort studies) that reported microvascular transfer of an upper-extremity fillet flap to reconstruct defects created by forequarter amputation, interscapulothoracic amputation, scapulothoracic amputation, or shoulder disarticulation. Eligible flaps included forearm, arm, hand, or composite designs, with or without bone (osteomyocutaneous variants). We excluded (i) pedicled fillet flaps without microvascular anastomosis, (ii) lower-extremity fillet flaps, (iii) animal/cadaveric studies, and (iv) reports without extractable outcome data for flap survival or postoperative morbidity.

### Information sources and search strategy

PubMed, Scopus, and Embase were systematically searched from database inception (1987) through 6 January 2025. Database-specific search strategies were developed to identify studies describing fillet flap or “spare parts” reconstruction in the setting of high-level upper-extremity amputation. In PubMed and Embase, controlled vocabulary was used where appropriate in combination with title/abstract terms; Scopus searches were conducted using TITLE-ABS-KEY fields.

Search strings combined (1) fillet-related terms (“fillet flap,” “free fillet,” “spare parts,” “spare-part”) and free tissue transfer terms (“free flap,” “free tissue transfer”) with (2) high-level upper-extremity amputation concepts, including forequarter, interscapulothoracic, scapulothoracic, shoulder disarticulation, shoulder girdle, upper-extremity amputation, major upper limb amputation, and high-level amputation. Additional anatomic modifiers (shoulder, thoracic, chest wall) were incorporated to improve sensitivity.

Results were restricted to English-language publications when supported by database functionality. The reference lists of all included studies were manually screened to identify additional eligible reports.

### Study selection and PRISMA flow

**Fig. 1:**
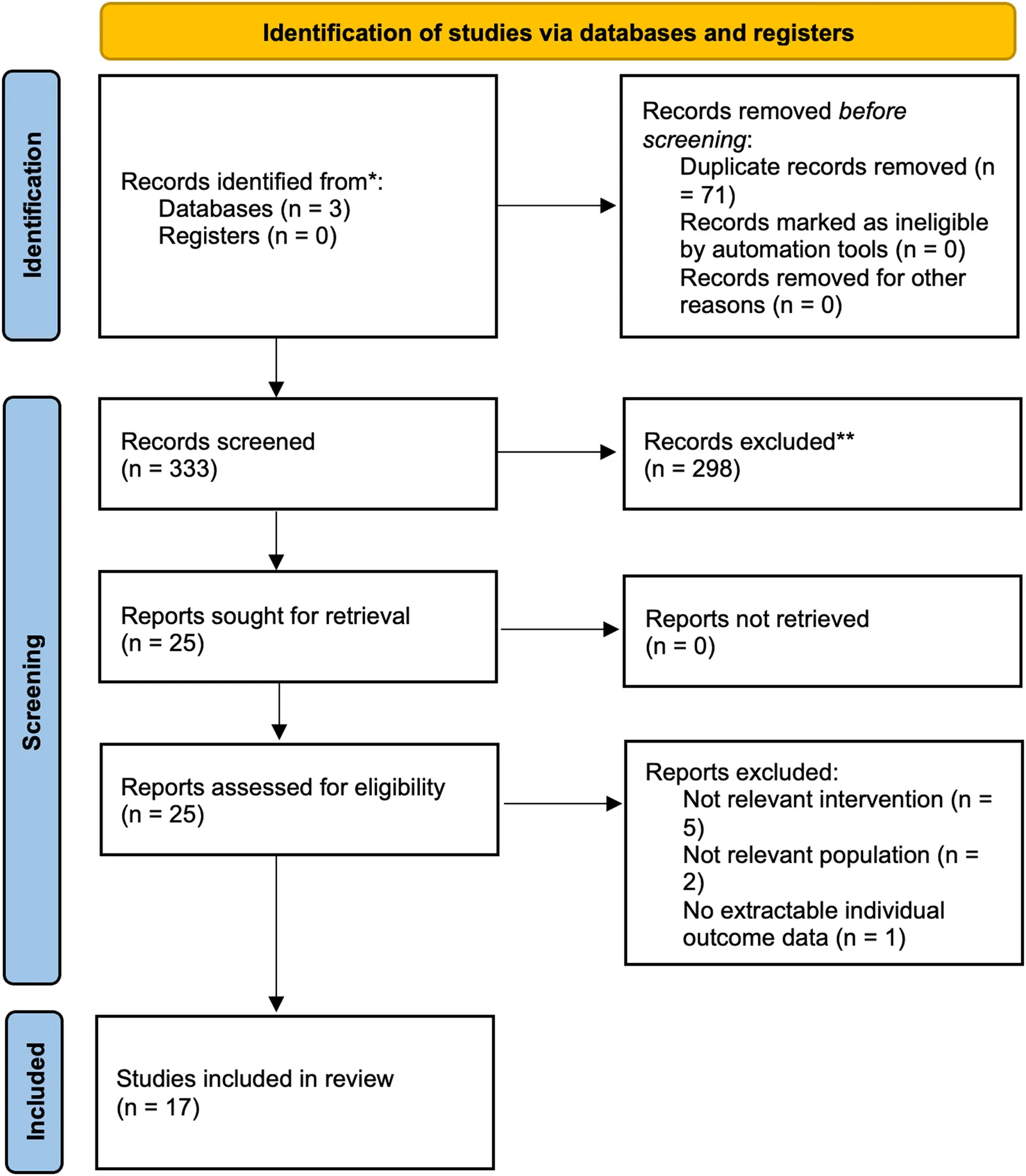
PRISMA flow diagram depicting the study identification, screening, eligibility assessment, and final inclusion process for the systematic review.

Searches of three databases (PubMed, Scopus, and Embase) identified 404 records. After removal of 71 duplicate records, 333 unique records remained and were screened by title and abstract, of which 298 were excluded. Twenty-five reports were sought for retrieval, all of which were obtained and assessed for full-text eligibility. At full-text review, eight reports were excluded (not relevant intervention, n = 5; not relevant population, n = 2; no extractable individual outcome data, n = 1), resulting in 17 studies included in the final review. The study selection process is summarized in the PRISMA flow diagram (Fig. 1).

### Data extraction and outcomes

Data were extracted into a standardized form, including: publication year and country; patient demographics; pathology; prior therapy (radiation and/or chemotherapy); comorbidities; amputation type; defect region(s); flap type and composition; follow-up; and postoperative outcomes. Primary outcomes were complete flap survival, any postoperative complication, and reoperation. Secondary outcomes included partial distal necrosis and complication subtypes (necrosis, thrombosis, dehiscence, infection, seroma, and other reported events).

### Methodological quality and certainty of evidence

Given that the available literature consisted predominantly of case reports and small case series, methodological quality appraisal was performed using the Joanna Briggs Institute (JBI) critical appraisal checklists for case reports and case series. These tools were selected a priori to account for the anticipated predominance of uncontrolled observational designs.

**Fig. 2:**
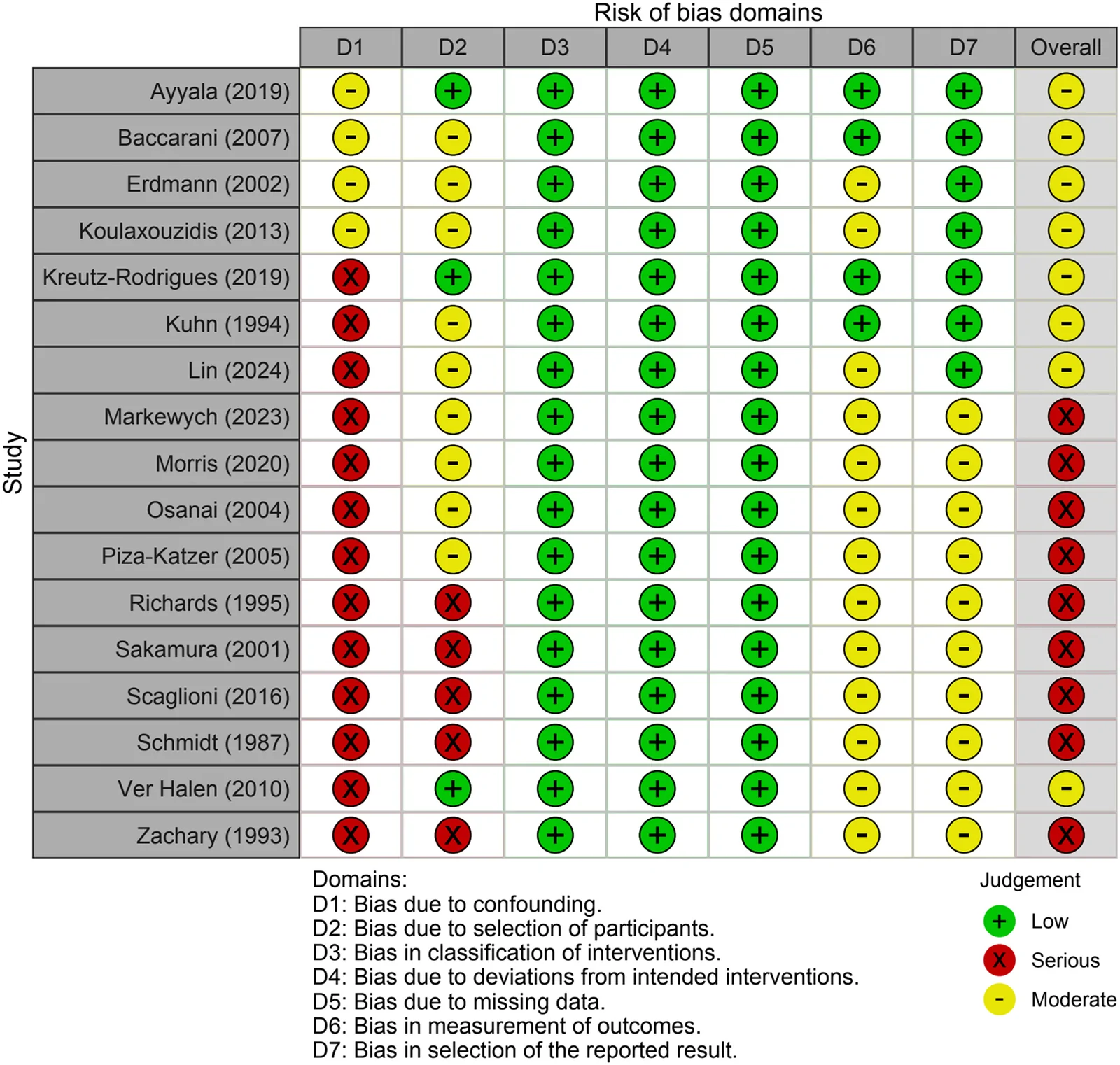
Risk of bias assessment of the included studies across seven domains using the ROBINS-I tool, categorized as low (green plus), moderate (yellow minus), or serious (red cross) risk of bias.

Most included studies were retrospective case reports or small case series and were judged to be of low to moderate methodological quality. Common limitations included non-consecutive inclusion of patients, incomplete follow-up reporting, absence of standardized complication grading, and limited detail regarding patient selection and outcome assessment. Of the 17 included studies, 9 were rated as having a serious overall risk of bias and 8 as moderate, with none assessed as low risk. A visualization of the risk of bias analysis is presented in Fig. 2.

Overall certainty of evidence for each primary outcome was summarized using GRADE principles. As uncontrolled observational designs begin at low certainty, evidence was further downgraded where appropriate for risk of bias, inconsistency, indirectness, and imprecision.

### Statistical analysis

Outcomes were pooled at the study level. For each included study, events and denominators were aggregated across reported patients (when applicable). Because several outcomes had extreme proportions and sparse events, random-effects meta-analysis of proportions used a generalized linear mixed model (GLMM) with a logit link and random intercept. Between-study heterogeneity was summarized with τ (logit-scale standard deviation) and 95% prediction intervals (PI). For robustness, sensitivity analyses for primary outcomes used inverse-variance random-effects pooling with Hartung–Knapp adjustment and continuity correction for zero cells, additionally reporting Q, I², and τ².

## Results

### Included studies and patient characteristics

Seventeen studies comprising 44 cases were included (Table 1). Publications spanned 1987–2024, with median publication year 2007 (IQR 2001–2019). Most reports originated from the United States (10/17), with additional studies from Japan (2/17) and single studies from Italy, Germany, Austria, the United Kingdom, and Switzerland.

**Table 1:** Study-level characteristics of included studies of free fillet upper-extremity flap (FFUE) reconstruction after forequarter-level amputation.

**Study**	**Cases (n)**	**Patient characteristics**	**Diagnosis**	**Amputation type**	**Flap design**	**Outcomes**
Ayyala et al.^5^	2	Age, mean (range): 43.5 (30–57) Sex (M/F): 1/1 Side (L/R/DNS): L=1, R=1 Prior therapy: RT=1, DNS=1 Comorbidity: 0/2	OS(1), Sarc(1)	Forequarter(2)	Forearm(2) Comp: FC-sub(1), MC(1)	Survival: complete 2/2 Complications: 0 Reoperation: 0/2 Follow-up (mo): DNS
Baccarani et al.^6^	1	Age, mean (range): 67 (67–67) Sex (M/F): 0/1 Side (L/R/DNS): R=1 Prior therapy: DNS=1 Comorbidity: 0/1	Sarc(1)	Shoulder disarticulation(1)	Forearm(1) Comp: MC(1)	Survival: complete 1/1 Complications: 0 Reoperation: 0/1 Follow-up (mo): DNS
Erdmann et al.^7^	1	Age, mean (range): 72 (72–72) Sex (M/F): 0/1 Side (L/R/DNS): R=1 Prior therapy: DNS=1 Comorbidity: 0/1	Histiocytoma(1)	Shoulder disarticulation(1)	Arm(1) Comp: MC(1)	Survival: complete 1/1 Complications: 0 Reoperation: 0/1 Follow-up (mo): DNS
Koulaxouzidis et al.^8^	4	Age, mean (range): 58.8 (46–73) Sex (M/F): 1/3 Side (L/R/DNS): L=2, R=2 Prior therapy: RT+CT=1, RT=2 Comorbidity: 2/4	Sarc(2), Lobular Breast Ca(1), Trauma(1)	Interscapulothoracic(4)	Composite elbow(4) Comp: OMC(4)	Survival: complete 3/4; partial 1 Complications: 3; Necrosis(2), Dehiscence(1), Delayed wound healing(1), Infection(1), Recurrence(1), Seroma(1), Thrombosis(1) Reoperation: 3/4 Follow-up (mo): 78.0 (24–132); 2 DNS
Kreutz-Rodrigues et al.^9^	5	Age, mean (range): 52.8 (21–65) Sex (M/F): 3/2 Side (L/R/DNS): L=1, R=4 Prior therapy: RT+CT=1, CT=1 Comorbidity: 4/5	CS(2), Fibrosarc(1), Liposarc(1), Sarc(1)	Forequarter(5)	Arm+forearm(5) Comp: FC-sup(2), MC(2), FC-sub(1)	Survival: complete 5/5 Complications: 1; Bleeding/hematoma(1), Dehiscence(1), Thrombosis(1) Reoperation: 1/5 Follow-up (mo): 12.2 (3.1–54.7)
Kuhn et al.^10^	1	Age, mean (range): 21 (21–21) Sex (M/F): 1/0 Side (L/R/DNS): R=1 Prior therapy: RT=1 Comorbidity: 0/1	Desmoid(1)	Forequarter(1)	Extended forearm OMC(1) Comp: OMC(1)	Survival: complete 1/1 Complications: 0 Reoperation: 0/1 Follow-up (mo): 12
Lin et al.^11^	2	Age, mean (range): 68.5 (67–70) Sex (M/F): 2/0 Side (L/R/DNS): R=2 Prior therapy: RT=1, DNS=1 Comorbidity: 0/2	Sarc(2)	Forequarter(2)	Arm(2) Comp: MC(2)	Survival: complete 2/2 Complications: 1; Necrosis(1) Reoperation: 1/2 Follow-up (mo): 11; 1 DNS
Markewych et al.^12^	1	Age, mean (range): 79 (79–79) Sex (M/F): 0/1 Side (L/R/DNS): L=1 Prior therapy: RT=1 Comorbidity: 1/1	CS(1)	Forequarter(1)	Forearm(1) Comp: MC(1)	Survival: complete 1/1 Complications: 0 Reoperation: 0/1 Follow-up (mo): 4
Morris et al.^13^	1	Age, mean (range): 60 (60–60) Sex (M/F): 1/0 Side (L/R/DNS): L=1 Prior therapy: DNS=1 Comorbidity: 0/1	Sarc(1)	Forequarter(1)	Forearm(1) Comp: MC(1)	Survival: complete 1/1 Complications: 1; Infection(1) Reoperation: 1/1 Follow-up (mo): 1
Osanai et al.^14^	2	Age, mean (range): 36 (16–56) Sex (M/F): 1/1 Side (L/R/DNS): R=2 Prior therapy: RT=1, CT=1 Comorbidity: 0/2	Cystosarcoma(1), OS(1)	Forequarter(2)	Composite elbow(2) Comp: OMC(2)	Survival: complete 2/2 Complications: 0 Reoperation: 0/2 Follow-up (mo): 8.0 (6–10)
Piza-Katzer et al.^15^	1	Age, mean (range): 54 (54–54) Sex (M/F): 0/1 Side (L/R/DNS): R=1 Prior therapy: RT+CT=1 Comorbidity: 0/1	Ductal Ca(1)	Forequarter(1)	Arm+forearm(1) Comp: FC-sub(1)	Survival: complete 1/1 Complications: 1; Necrosis(1) Reoperation: 1/1 Follow-up (mo): 9
Richards et al.^16^	2	Age, mean (range): 60.5 (57–64) Sex (M/F): 2/0 Side (L/R/DNS): L=1, R=1 Prior therapy: DNS=2 Comorbidity: 0/2	BCC(1), SCC(1)	Forequarter(2)	Forearm(2) Comp: FC-sub(2)	Survival: complete 2/2 Complications: 0 Reoperation: 0/2 Follow-up (mo): 30.0 (24–36)
Sakamura et al.^17^	1	Age, mean (range): 57 (57–57) Sex (M/F): 0/1 Side (L/R/DNS): L=1 Prior therapy: CT=1 Comorbidity: 0/1	Breast Ca(1)	Forequarter(1)	Forearm+hand(1) Comp: FC-sub(1)	Survival: complete 1/1 Complications: 1; Necrosis(1) Reoperation: 1/1 Follow-up (mo): 22
Scaglioni et al.^18^	1	Age, mean (range): 41 (41–41) Sex (M/F): 0/1 Side (L/R/DNS): L=1 Prior therapy: RT+CT=1 Comorbidity: 0/1	Desmoid(1)	Forequarter(1)	Forearm(1) Comp: FC-sub(1)	Survival: complete 1/1 Complications: 1; Dehiscence(1), Necrosis(1) Reoperation: 1/1 Follow-up (mo): 19
Schmidt et al.^19^	1	Age, mean (range): 34 (34–34) Sex (M/F): 1/0 Side (L/R/DNS): L=1 Prior therapy: RT=1 Comorbidity: 1/1	CS (secondary)(1)	Forequarter(1)	Forearm(1) Comp: MC(1)	Survival: complete 1/1 Complications: 1; Respiratory insufficiency(1) Reoperation: 0/1 Follow-up (mo): 12
Ver Halen et al.^20^	16	Age, mean (range): 57.9 (33–78) Sex (M/F): 14/2 Side (L/R/DNS): L=1, DNS=15 Prior therapy: RT+CT=12, CT=4 Comorbidity: 0/16	MFH(5), Melanoma(4), CS(2), BCC(1), DFSP(1), OS(1), SCC(1), Sarc(1)	Forequarter(16)	Forearm(11), Arm+forearm(4), Arm(1) Comp: MC(16)	Survival: complete 16/16 Complications: 3; Anastomosis disruption(1), Myocardial infarction(1), Necrosis(1), Seroma(1), Thrombosis(1) Reoperation: 2/16 Follow-up (mo): 6.0 (0.3–48)
Zachary et al.^21^	2	Age, mean (range): 75.5 (70–81) Sex (M/F): 1/1 Side (L/R/DNS): R=2 Prior therapy: RT=2 Comorbidity: 0/2	CS(1), Large-cell Ca(1)	Forequarter(2)	Forearm(2) Comp: FC-sub(2)	Survival: complete 2/2 Complications: 0 Reoperation: 0/2 Follow-up (mo): 6.0 (6–6)

Abbreviations: BCC = basal cell carcinoma; Breast Ca = breast cancer; CS = chondrosarcoma; CT = chemotherapy; DFSP = dermatofibrosarcoma protuberans; DNS = data not specified; FC-sub = fasciocutaneous (subfascial); FC-sup = fasciocutaneous (suprafascial); FFUE = free fillet upper-extremity flap; MC = musculocutaneous; MFH = malignant fibrous histiocytoma; OMC = osteomyocutaneous; OS = osteosarcoma; RT = radiotherapy; Sarc = sarcoma.

Patients were predominantly male (28/44, 63.6%), with mean age 56.3 years (SD 16.1; range 16–81). Follow-up was reported in 37/44 cases (88.6%), with median follow-up 8 months (IQR 5–19) and mean 16.0 months (SD 23.2). A detailed summary of study-level and patient-level characteristics is provided in Table 2.

**Table 2:** Included evidence base and patient demographics.

Domain	Summary
Studies / cases	17 studies; 44 cases
Publication years	Median 2007 (IQR 2001–2019); range 1987–2024
Countries (studies)	USA 10; Japan 2; Italy 1; Germany 1; Austria 1; UK 1; Switzerland 1
Age (years)	Mean 56.34 ±16.06; median 58 (IQR 46.75–70); range 16–81
Sex	Male 28 (63.6%); female 16 (36.4%)
Follow-up	Reported in 37 (88.6%); median 8 months (IQR 5–19); mean 15.99 ±23.20 months

This table summarizes the overall evidence base and pooled patient demographics for the included studies, detailing metrics such as study counts, publication years, country distributions, age, sex, and follow-up durations.

### Indications, prior therapy, and comorbidity

Underlying pathology was predominantly malignant. Soft tissue sarcoma/mesenchymal tumors accounted for 47.7% (21/44) and bone sarcomas 22.7% (10/44); carcinomas and melanomas comprised 18.2% (8/44) and 9.1% (4/44), respectively; one case followed trauma (2.3%).

Prior multimodal therapy was common: 36.4% had received both radiotherapy and chemotherapy, 22.7% radiotherapy alone, and 15.9% chemotherapy alone; only 9.1% had no prior therapy reported. Comorbidity reporting was inconsistent; where stated, 18.2% had at least one comorbidity (cardiovascular disease 11.4%, diabetes 6.8%, chronic lung disease 4.5%, obesity 4.5%, chronic kidney disease 2.3%). A comprehensive summary of underlying diagnoses, prior oncologic treatment, and reported comorbidities is provided in Table 3.

**Table 3:** Pathology, prior treatment, comorbidities, and amputation type.

Variable	Category	Events (n/N)	Rate (%)	95% CI
**Diagnosis**	Soft tissue sarcoma/mesenchymal	21/44	47.7	33.8–62.1
	Bone sarcoma	10/44	22.7	12.8–37.0
	Carcinoma	8/44	18.2	9.5–32.0
	Melanoma	4/44	9.1	3.6–21.2
	Trauma / non-neoplastic	1/44	2.3	0.4–11.8
**Prior therapy**	Radiotherapy only	10/44	22.7	12.8–37.0
	Chemotherapy only	7/44	15.9	7.9–29.4
	Both radiation and chemotherapy	16/44	36.4	23.8–51.1
	None	4/44	9.1	3.6–21.2
	Not specified (DNS)	7/44	15.9	7.9–29.4
**Comorbidities**	Any comorbidity reported	8/44	18.2	9.5–32.0
	None reported	36/44	81.8	68.0–90.5
	Chronic lymphoedema	2/44	4.5	1.3–15.1
	Hypertension	2/44	4.5	1.3–15.1
	Diabetes Mellitus	1/44	2.3	0.4–11.8
	Cardiovascular disease	1/44	2.3	0.4–11.8
	Radiation-induced brachial plexopathy	1/44	2.3	0.4–11.8
	Multiple hereditary exostoses	1/44	2.3	0.4–11.8
**Amputation type**	Forequarter	38/44	86.4	73.3–93.6
	Interscapulothoracic	4/44	9.1	3.6–21.2
	Shoulder disarticulation	2/44	4.5	1.3–15.1

### Amputation type, defect regions, and flap design

Most reconstructions followed forequarter amputation (86.4%, 38/44), with smaller numbers after interscapulothoracic amputation (9.1%, 4/44) and shoulder disarticulation (4.5%, 2/44). Defects most frequently involved the chest wall/thorax (47.7%), shoulder region (45.5%), and axilla (34.1%); multiple regions were often involved (non–mutually exclusive categories).

FFUE flap design varied: free fillet forearm flaps were most common (50.0%), followed by composite arm+forearm (22.7%), composite elbow designs (13.6%), and arm-only flaps (9.1%), with rare variants including forearm+hand and extended osseomyocutaneous forearm constructs. Most flaps were musculocutaneous (59.1%), with smaller proportions of fasciocutaneous (25.0% combined suprafascial/subfascial) and osteomyocutaneous designs (15.9%). Detailed distributions of amputation type, defect location, and flap characteristics are summarized in Table 4.

**Table 4:** Defect and flap characteristics.

Variable	Category	Events (n/N)	Rate (%)	95% CI
Defect region(s)*	Chest wall/thorax	21/44	47.7	33.8–62.1
	Shoulder region	20/44	45.5	31.7–59.9
	Axilla	15/44	34.1	21.9–48.9
	Upper arm	3/44	6.8	2.3–18.2
	Elbow	1/44	2.3	0.4–11.8
	Not specified (DNS)	5/44	11.4	—
**FFUE design**	Forearm	22/44	50.0	35.8–64.2
	Arm + forearm	10/44	22.7	12.8–37.0
	Composite elbow	6/44	13.6	6.4–26.7
	Arm	4/44	9.1	3.6–21.2
	Forearm + hand	1/44	2.3	0.4–11.8
	Extended forearm osseomyocutaneous	1/44	2.3	0.4–11.8
**Flap composition**	Musculocutaneous	26/44	59.1	44.4–72.3
	Fasciocutaneous (subfascial)	9/44	20.5	11.2–34.5
	Osteomyocutaneous	7/44	15.9	7.9–29.4
	Fasciocutaneous (suprafascial)	2/44	4.5	1.3–15.1

*Non–mutually exclusive; defects often involved multiple regions.

Across studies, intraoperative details (recipient vessels, ischemia time, anticoagulation, monitoring strategy) were variably reported and could not be pooled. Nevertheless, technique-oriented reports describe using large-caliber recipient vessels within the subclavian/axillary system and the brachial artery as inflow for volar forearm fillet flaps, and emphasize back-table harvest with a clean instrument set for oncologic hygiene.

Across the 17 included studies, the fillet flap was consistently harvested from tissue distal to the planned oncologic resection margin. However, none of the studies described a standardized protocol to evaluate the harvested flap tissue for occult malignancy before transfer. No study reported the routine use of frozen-section analysis, dedicated pathological assessment of the flap tissue, or other specific screening methods beyond standard oncologic surgical planning. Instead, authors generally implied oncologic safety by selecting tissue outside the planned resection margins and grossly uninvolved by tumor.

### Primary outcomes

Complete flap survival occurred in 43/44 cases (97.7%); one case (2.3%) had partial distal necrosis and there were no total flap losses in the extracted dataset.

In GLMM random-effects meta-analysis, pooled complete survival was 97.0% (95% CI 88.8–99.3) with τ=0.75 and 95% PI 88.3–99.3. Any complication occurred in 13/44 cases (29.5%), and reoperation in 11/44 (25.0%). Pooled complication and reoperation rates were 31.2% (95% CI 18.7–47.3; τ=0.90; PI 7.2–72.8) and 26.0% (95% CI 14.6–42.0; τ=1.01; PI 4.6–71.9), respectively (Table 5).

**Table 5:** Random-effects meta-analysis of primary outcomes and key complication subtypes (GLMM).

Outcome	Studies (k)	Events (n/N)	Rate (%)	Pooled proportion % (95% CI)	τ	95% Prediction interval
**Complete flap survival**	17	43/44	97.7	97.0 (88.8–99.3)	0.75	88.3–99.3
**Any complication**	17	13/44	29.5	31.2 (18.7–47.3)	0.90	7.2–72.8
**Reoperation**	17	11/44	25.0	26.0 (14.6–42.0)	1.01	4.6–71.9
**Necrosis**	17	7/44	15.9	16.1 (7.7–30.7)	1.08	2.2–62.3
**Thrombosis**	17	3/44	6.8	6.8 (2.5–17.3)	0.56	1.7–24.3
**Dehiscence**	17	3/44	6.8	6.9 (2.5–17.9)	0.80	1.2–31.3
**Infection**	17	2/44	4.5	4.9 (1.5–14.7)	0.88	0.6–31.4
**Seroma**	17	2/44	4.5	4.8 (1.5–14.2)	0.60	1.1–18.2

Prediction intervals reflect expected variability in new settings.

Sensitivity analyses using inverse-variance pooling with Hartung–Knapp adjustment produced similar pooled estimates for complications (32.1%) and reoperation (26.9%), with I² reported as 0% for these outcomes, an estimate that should be interpreted cautiously in sparse-data meta-analysis. Given the predominance of single-patient case reports, heterogeneous outcome definitions, and inconsistent reporting across studies, these pooled estimates should be interpreted as descriptive summaries of the published literature rather than precise estimates of true clinical event rates.

### Complications

Among 44 cases, complication subtypes included necrosis (7), thrombosis (3), dehiscence (3), infection (2), seroma (2), bleeding/hematoma (1), recurrence (1), respiratory insufficiency (1), myocardial infarction (1), and delayed wound healing (1). Multiple complications could occur in the same patient. GLMM pooled complication subtype rates were: necrosis 16.1% (95% CI 7.7–30.7), thrombosis 6.8% (2.5–17.3), dehiscence 6.9% (2.5–17.9), infection 4.9% (1.5–14.7), and seroma 4.8% (1.5–14.2). A comprehensive summary of pooled primary outcomes (complete flap survival, overall complication, and reoperation rates) and key complication subtypes, including between-study heterogeneity (τ) and 95% prediction intervals, is presented in Table 5.

## Discussion

This review synthesizes the available evidence for free fillet of the upper extremity (FFUE) reconstruction following forequarter-level ablative surgery and provides pooled estimates of survival, complications, and reoperation. However, interpretation of the pooled estimates requires caution because the available literature consists almost entirely of heterogeneous case reports and small case series with inconsistent outcome definitions, variable follow-up, and non-standardized reporting. Consequently, the meta-analysis should be viewed primarily as a quantitative summary of published experience rather than definitive evidence of treatment effect. Three principal findings merit emphasis.

First, flap reliability appears strikingly high: the pooled estimate of complete survival was 97%, with no total flap losses reported in the extracted dataset. In a population characterized by extensive composite defects, frequent prior radiotherapy or chemotherapy, and physiologic stress, this suggests that FFUE transfer functions as a dependable option when conventional donor sites are undesirable, unavailable, or associated with unacceptable additional morbidity.

Second, complications (≈31%) and reoperations (≈26%) are not uncommon. These rates should not be interpreted as technical failure; rather, they likely reflect the baseline risk inherent to forequarter-level oncologic surgery, which often entails massive dead space, intrathoracic or pleural exposure, mesh reconstruction, and previously irradiated recipient beds. The predominance of musculocutaneous designs (59%) in the pooled dataset aligns with reconstructive principles for complex chest wall and shoulder girdle defects, where vascularized muscle is advantageous for obliterating dead space, protecting vascular stumps and prosthetic material, and mitigating infection or wound breakdown.

Third, the overall quality of evidence remains limited and reporting inconsistent. Key intraoperative variables, including recipient vessel selection, ischemia time, anticoagulation strategy, and monitoring protocols, in addition to patient-centered outcomes such as pain, prosthetic integration, and quality of life were inconsistently documented, precluding meaningful moderator analyses and limiting interpretability of pooled estimates.

Several biologic and technical features plausibly contribute to the high observed survival rate. FFUE harvest provides large-caliber donor vessels derived from the brachial, radial, or ulnar systems, typically with generous venous drainage.^22^ Recipient options after forequarter amputation frequently include the subclavian or axillary vessels, which also offer substantial luminal diameter. Large-caliber arterial and venous anastomoses may reduce the risk of technical thrombosis relative to smaller perforator-based reconstructions.^23^ In addition, these complex reconstructions are generally undertaken in tertiary referral centers by experienced microsurgeons, introducing a probable “center effect.” At the same time, publication bias almost certainly inflates apparent success; failures and near-failures are less likely to be reported, particularly in rare oncologic scenarios where reconstruction is individualized and highly context dependent.

Necrosis emerged as the most frequent complication (pooled ≈16%), and likely represents a spectrum rather than a single entity. Events may include marginal skin paddle ischemia in distal zones of long fillet designs, partial flap compromise necessitating debridement, or wound-edge necrosis related to a previously irradiated and poorly vascularized recipient bed. Importantly, necrosis did not translate into reported total flap loss in the analyzed cases, suggesting that most events were partial and salvageable. From a practical standpoint, surgeons should anticipate staged debridement or contouring when large skin paddles span multiple anatomic subregions (e.g., shoulder, axilla, and chest wall). Technical considerations that may mitigate risk include avoiding reliance on the most distal angiosomes, trimming to clearly perfused tissue at inset, minimizing tension and shear, and employing well-vascularized muscle to support coverage over mesh or exposed mediastinal or vascular structures.

Although comparative effectiveness between FFUE and conventional free flaps cannot be determined from case reports and small series, several rational indications for a “spare-parts” approach emerge. Avoidance of additional donor-site morbidity is particularly relevant in frail patients, those with limited residual limb function, or individuals at elevated risk of wound complications due to malnutrition, immunosuppression, or prior radiation. FFUE also offers the ability to transfer extraordinary volumes of skin and muscle without donor-site closure constraints, an important advantage when forequarter resection creates extensive surface loss and deep dead space. Immediate durable closure during the same anesthetic may reduce prolonged open-wound management and facilitate timely initiation or resumption of adjuvant oncologic therapy.^24^ Finally, the technique uniquely permits integration with targeted muscle reinnervation (TMR) or other nerve procedures, potentially addressing neuroma and phantom limb pain while preserving options for advanced myoelectric prosthetics in high-level amputees.^25^ This combined oncologic–reconstructive–functional paradigm represents an emerging direction that warrants prospective evaluation.

Given the rarity of FFUE reconstruction, progress in this field depends on improved and standardized reporting. Future reports and multicenter registries should consistently document tumor histology and margin status; prior radiotherapy or chemotherapy and timing; detailed defect mapping including subunits involved, prosthetic or mesh use, and pleural or lung exposure; flap design characteristics (segments included, muscle components, pedicle choice, inclusion of bone or hand elements); recipient vessels, ischemia time, anticoagulation regimen, and monitoring protocols; complications graded with a standardized system; and patient-centered outcomes such as pain scores, neuroma management, prosthetic fitting and use, satisfaction, and validated quality-of-life measures. Such uniformity would enable meaningful pooled analyses, identification of predictors of adverse events, and more accurate counseling of patients.

This review has several strengths. Although FFUE reconstruction is an uncommon procedure described almost exclusively in small observational reports, pooling the available evidence allows a broader assessment of reconstructive outcomes than any individual publication can provide. The resulting estimates help contextualize the consistently high flap survival reported across diverse institutions while simultaneously highlighting the uncertainty surrounding complications, reoperations, and long-term functional outcomes. By identifying these knowledge gaps, this review establishes a foundation for future collaborative studies and standardized reporting in this rare reconstructive population.

This review also has important limitations. First, the evidence base consists almost entirely of uncontrolled case reports and small case series, subject to substantial selection and publication bias. Second, methodological appraisal tools can characterize but not eliminate these biases. Third, quantitative synthesis is constrained by small-study effects and sparse events; prediction intervals for complications and reoperations were broad, indicating that real-world rates may vary considerably by institutional expertise and case complexity. Fourth, follow-up duration and long-term outcomes were inconsistently reported, precluding robust synthesis of oncologic control, durability of reconstruction, pain trajectories, and functional recovery. Fifth, the included studies did not report standardized methods for confirming the oncologic suitability of harvested fillet flap tissue beyond adherence to planned resection margins, limiting assessment of the risk of occult malignancy within transferred tissue. Accordingly, while FFUE appears to be a highly reliable reconstructive strategy in selected patients undergoing forequarter-level ablation, conclusions regarding comparative effectiveness and long-term benefit must remain cautious pending higher-quality, collaborative data.

## Conclusions

In the published literature, FFUE reconstruction after forequarter-level ablative surgery demonstrates high survival with no reported total flap losses in the extracted dataset in clinical practice. Complications, particularly necrosis, and reoperations are common but appear acceptable given the extreme baseline risk profile. Standardized reporting and multi-institutional registries are needed to refine operative strategy and to evaluate patient-centered outcomes, including pain control and prosthetic function.

## Ethical approval

Not required.

## Funding

This research received no external funding.

## Declaration of competing interest

The authors declare no conflicts of interest.
